# Early local recurrence of well-differentiated liposarcoma of the hypopharynx in a young adult: a case report and literature review

**DOI:** 10.3389/fonc.2026.1877159

**Published:** 2026-06-22

**Authors:** Yi Zhong, Long Chen, Suru Liu, Haiying Sun

**Affiliations:** 1 Department of Otorhinolaryngology, Union Hospital, Tongji Medical College, Huazhong University of Science and Technology, Wuhan, China; 2 Department of Neurosurgery, Union Hospital, Tongji Medical College, Huazhong University of Science and Technology, Wuhan, China; 3 Shanghai Sixth People’s Hospital Affiliated to Shanghai Jiao Tong University School, Shanghai, China

**Keywords:** case report, hypopharynx, laryngoscopy, mdm2 amplification, well-differentiated liposarcoma

## Abstract

Well-differentiated liposarcoma (WDLPS), also termed atypical lipomatous tumor, is a low-grade malignant adipocytic neoplasm characterized by indolent growth, limited metastatic potential, and a propensity for local recurrence. Primary WDLPS arising in the hypopharyngeal region is exceptionally rare, particularly in young adults, and may clinically mimic benign submucosal lesions. We report the case of a 24-year-old man who presented with a one-month history of persistent foreign body sensation in the throat. Flexible laryngoscopy revealed a smooth submucosal mass arising from the left hypopharyngeal region, and contrast-enhanced magnetic resonance imaging demonstrated a well-circumscribed enhancing lesion without cervical lymphadenopathy. The mass was excised transorally under general anesthesia. Histopathological examination showed mature adipocytic proliferation with atypical stromal cells, and immunohistochemistry demonstrated positivity for MDM2, CDK4, CD34, and p16. Fluorescence *in situ* hybridization confirmed MDM2 gene amplification, establishing the diagnosis of WDLPS. Although the early postoperative course was uneventful and initial follow-up showed satisfactory mucosal healing, serial laryngoscopic examinations at 2, 2.5, and 4 months after surgery revealed early local recurrence at the surgical site. This case highlights that hypopharyngeal WDLPS can occur in young adults and may recur early even after apparently complete transoral excision. Accurate diagnosis requires integration of histopathology, MDM2/CDK4 immunohistochemistry, and molecular confirmation of MDM2 amplification. Close endoscopic and radiological surveillance is essential for early detection of local recurrence.

## Introduction

Liposarcoma is among the most common malignant soft tissue tumours in adults; however, involvement of the head and neck region is uncommon, accounting for approximately 2–9% of all liposarcomas (1). The hypopharyngeal region is an extremely unusual site, with only isolated case reports and small case series described in the literature (2–5). According to the WHO Classification of Soft Tissue and Bone Tumours, atypical lipomatous tumour/well-differentiated liposarcoma (ALT/WDLPS) represents a low-grade adipocytic neoplasm characterized by slow growth, a propensity for local recurrence, and limited metastatic potential (6, 7).

## Case presentation

A 24-year-old male presented to our department with a one-month history of persistent foreign body sensation in the throat. The discomfort was localized to the left side, aggravated by swallowing, and was not associated with pain, hoarseness, dyspnea, or weight loss. He denied fever, night sweats, fatigue, or other constitutional symptoms. He had no relevant past medical history and no history of smoking or alcohol abuse. Prior to presentation, the patient had received empirical medical treatment, including proton pump inhibitors and anti-inflammatory medication, without significant symptomatic relief. The foreign body sensation was evaluated clinically based on the patient’s history and serial endoscopic findings; no validated symptom score or visual analog scale was prospectively recorded.

## Diagnostic assessment

**Figure 1:**
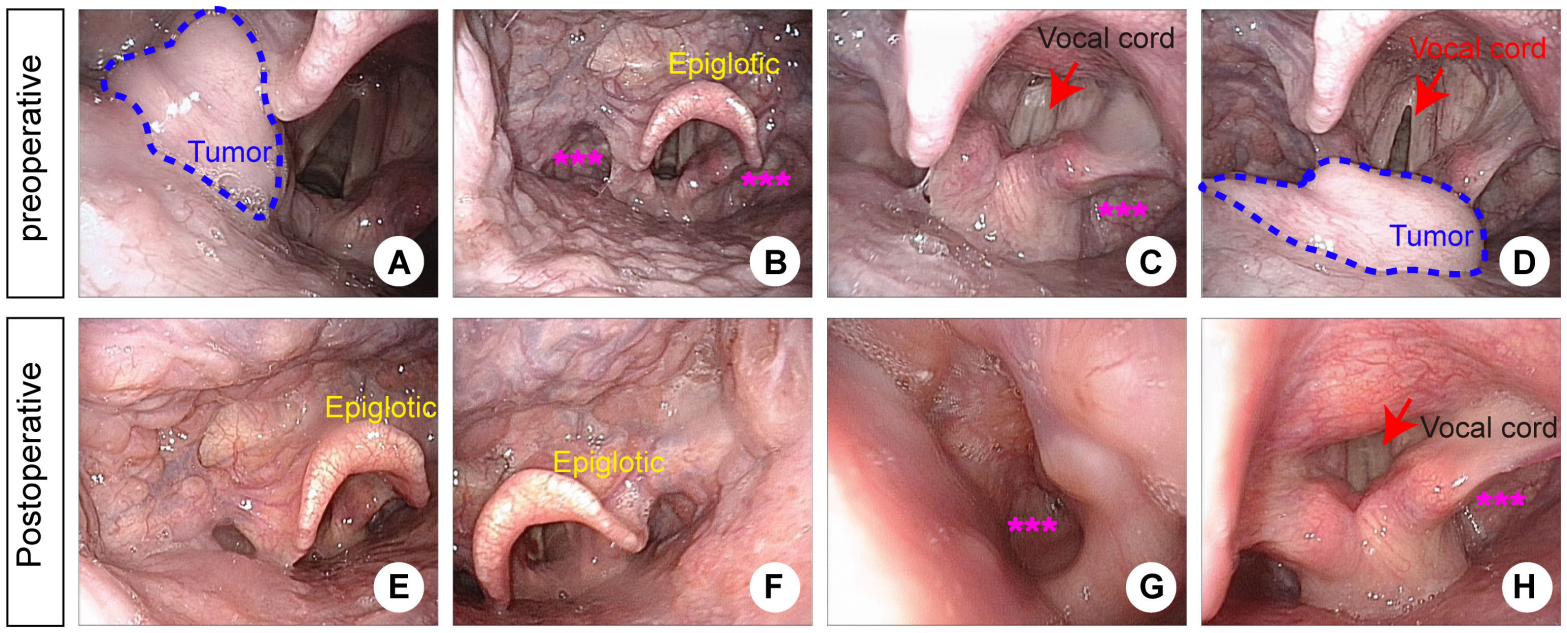
Flexible laryngoscopic findings. Preoperative views **(A–D)** demonstrated a smooth, submucosal, well-circumscribed mass in the left hypopharyngeal region, with the tumor outlined by blue dashed lines in representative views **(A, D)**. The epiglottis and vocal cords are labeled in representative views **(B, C)**. Postoperative views **(E–H)** showed removal of the mass, satisfactory mucosal healing, and clear visualization of the epiglottis and vocal cords. The overlying mucosa was intact, and no obvious ulceration or bleeding was observed.

**Figure 2:**
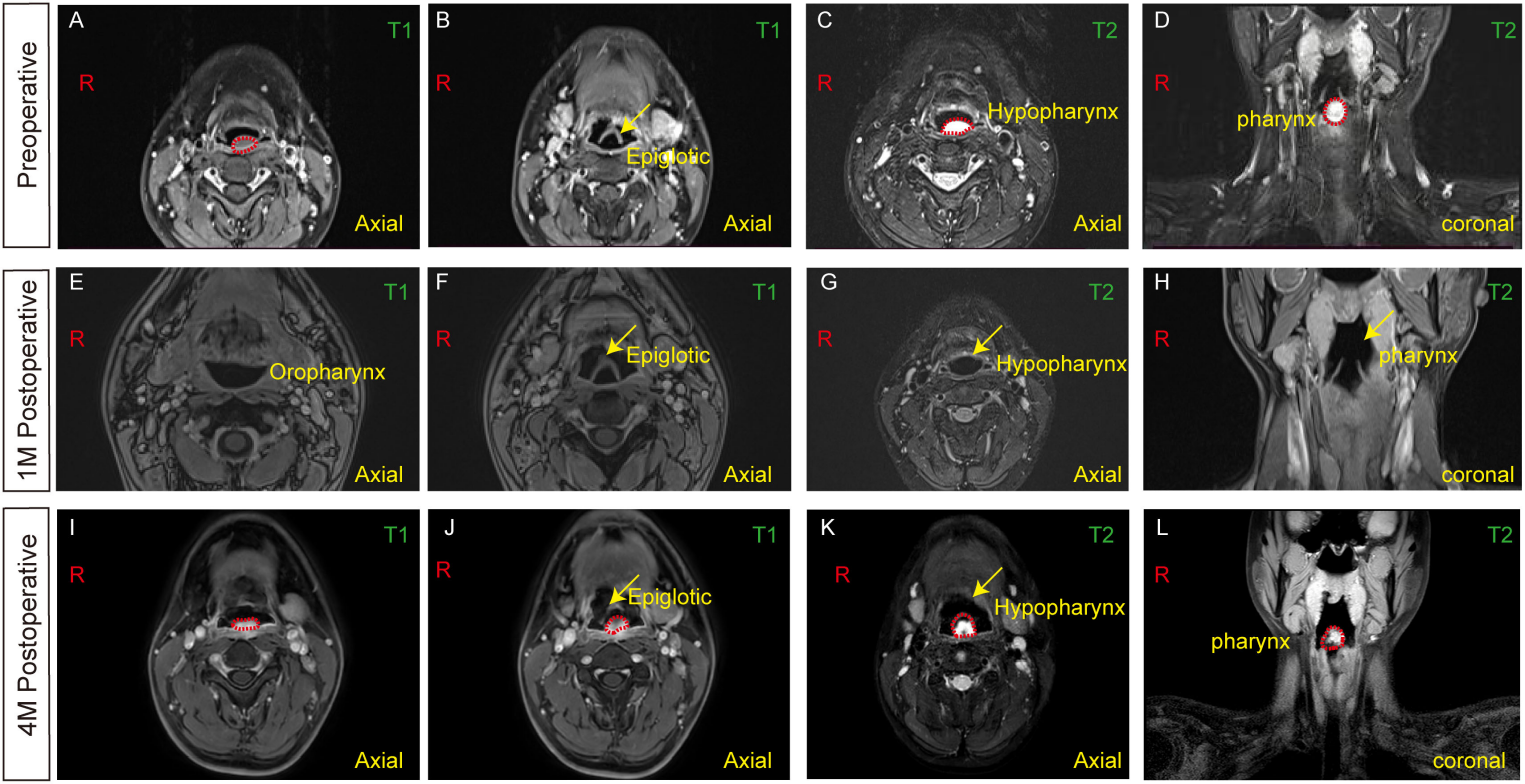
Preoperative and postoperative MRI findings. Preoperative MRI showed a well-circumscribed submucosal lesion in the left hypopharyngeal region. Axial T1-weighted images **(A, B)**, axial T2-weighted image **(C)**, and coronal T2-weighted image **(D)** demonstrated the lesion adjacent to the epiglottis and hypopharyngeal/pharyngeal region. One month after surgery, axial T1-weighted images **(E, F)**, axial T2-weighted image **(G)**, and coronal T2-weighted image **(H)** showed postoperative changes without definite residual tumor. Four months after surgery, axial T1-weighted images **(I, J)**, axial T2-weighted image **(K)**, and coronal T2-weighted image **(L)** demonstrated a newly detected enhancing lesion at the operative site, supporting early local recurrence.

**Figure 3:**
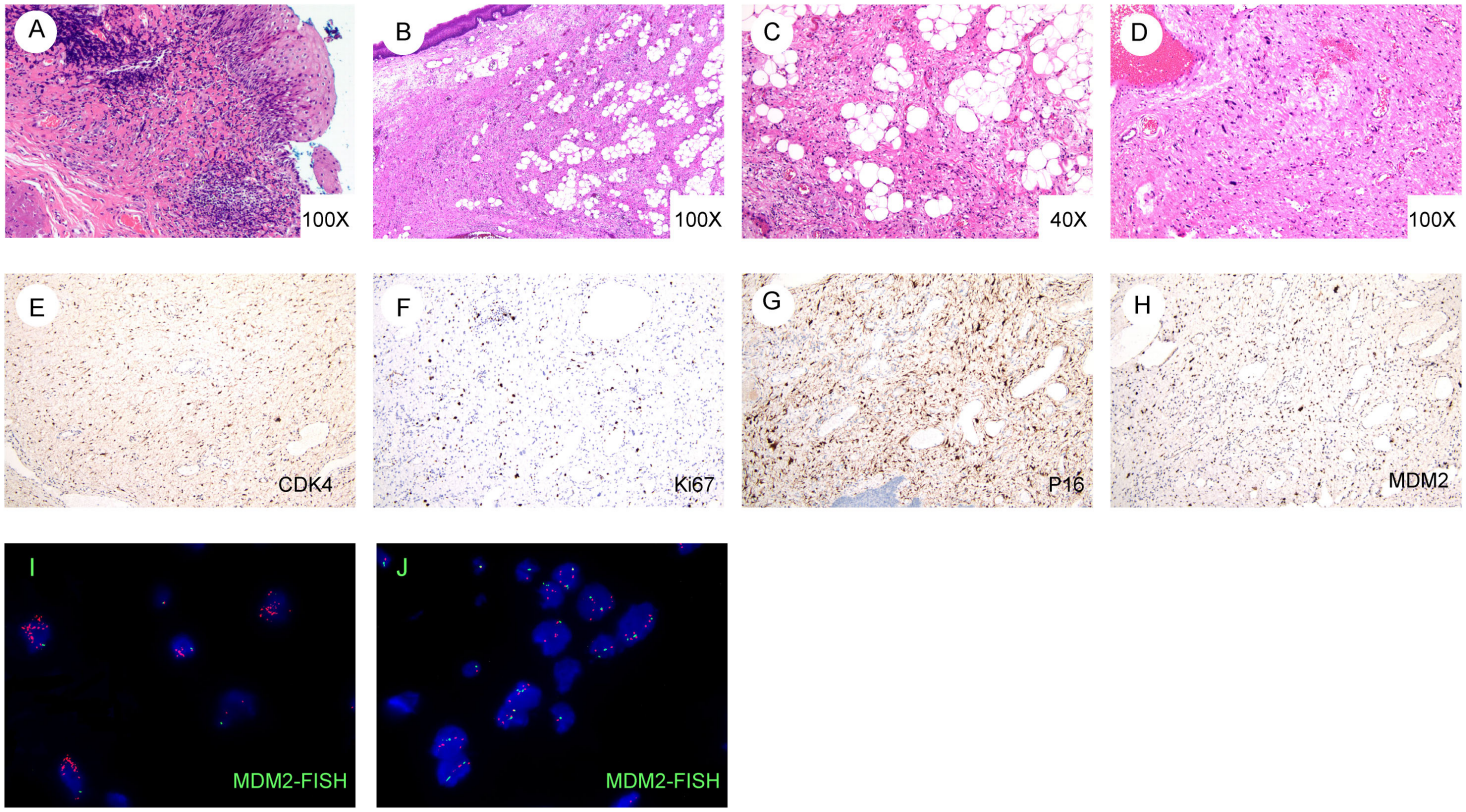
Histopathological, immunohistochemical, and molecular findings. Hematoxylin and eosin staining showed mucosa with chronic inflammatory cell infiltration and underlying adipocytic tumor components **(A, B)**. The tumor was composed of mature adipocytes admixed with atypical stromal cells and fibrous areas, consistent with well-differentiated liposarcoma/atypical lipomatous tumor **(C, D)**. Immunohistochemical staining showed nuclear positivity for CDK4 **(E)**, a low Ki-67 proliferation index **(F)**, positivity for p16 **(G)**, and nuclear positivity for MDM2 **(H)**. Fluorescence *in situ* hybridization demonstrated MDM2 gene amplification **(I, J)**, confirming the molecular diagnosis of well-differentiated liposarcoma.

Laryngoscopy revealed a smooth-surfaced submucosal mass in the left hypopharyngeal region (**Figure 1**). MRI demonstrated a well-circumscribed, homogeneously enhancing submucosal lesion measuring approximately 17 × 7 mm, without evidence of cervical lymphadenopathy (**Figures 2A–D**). Immunohistochemical analysis showed diffuse nuclear positivity for MDM2 and CDK4, positivity for CD34 and p16, focal positivity for S100 and desmin, and negative staining for SOX10, smooth muscle actin, and epithelial membrane antigen, with a Ki-67 proliferation index of approximately 5%. FISH confirmed MDM2 amplification, supporting the diagnosis of well-differentiated liposarcoma (**Figure 3**). Benign submucosal lesions were considered in the differential diagnosis. Lipoma was considered less likely because of the presence of atypical stromal cells and MDM2/CDK4 positivity. Schwannoma and neurofibroma were not supported by the immunophenotype, particularly SOX10 negativity and only focal S100 staining. Minor salivary gland tumors were unlikely because there was no epithelial tumor component and epithelial membrane antigen was negative. Fibroma was also excluded by the adipocytic morphology, atypical stromal cells, and molecular confirmation of MDM2 amplification.

## Therapeutic intervention

The patient underwent transoral endoscopic excision of the hypopharyngeal mass under general anesthesia. Intraoperatively, the lesion was exposed using direct laryngoscopy. The mass was located in the left hypopharyngeal region and appeared smooth, submucosal, well circumscribed, and encapsulated. A mucosal incision was made around the lesion, followed by careful submucosal dissection along the tumor capsule. The tumor was removed en bloc with an attempt to preserve an adequate mucosal and submucosal margin while maintaining pharyngolaryngeal function. No obvious invasion of adjacent structures was observed intraoperatively, and no major bleeding or airway-related complication occurred. Final histopathological examination confirmed well-differentiated liposarcoma with negative surgical margins.

## Follow-up and outcomes

**Figure 4:**
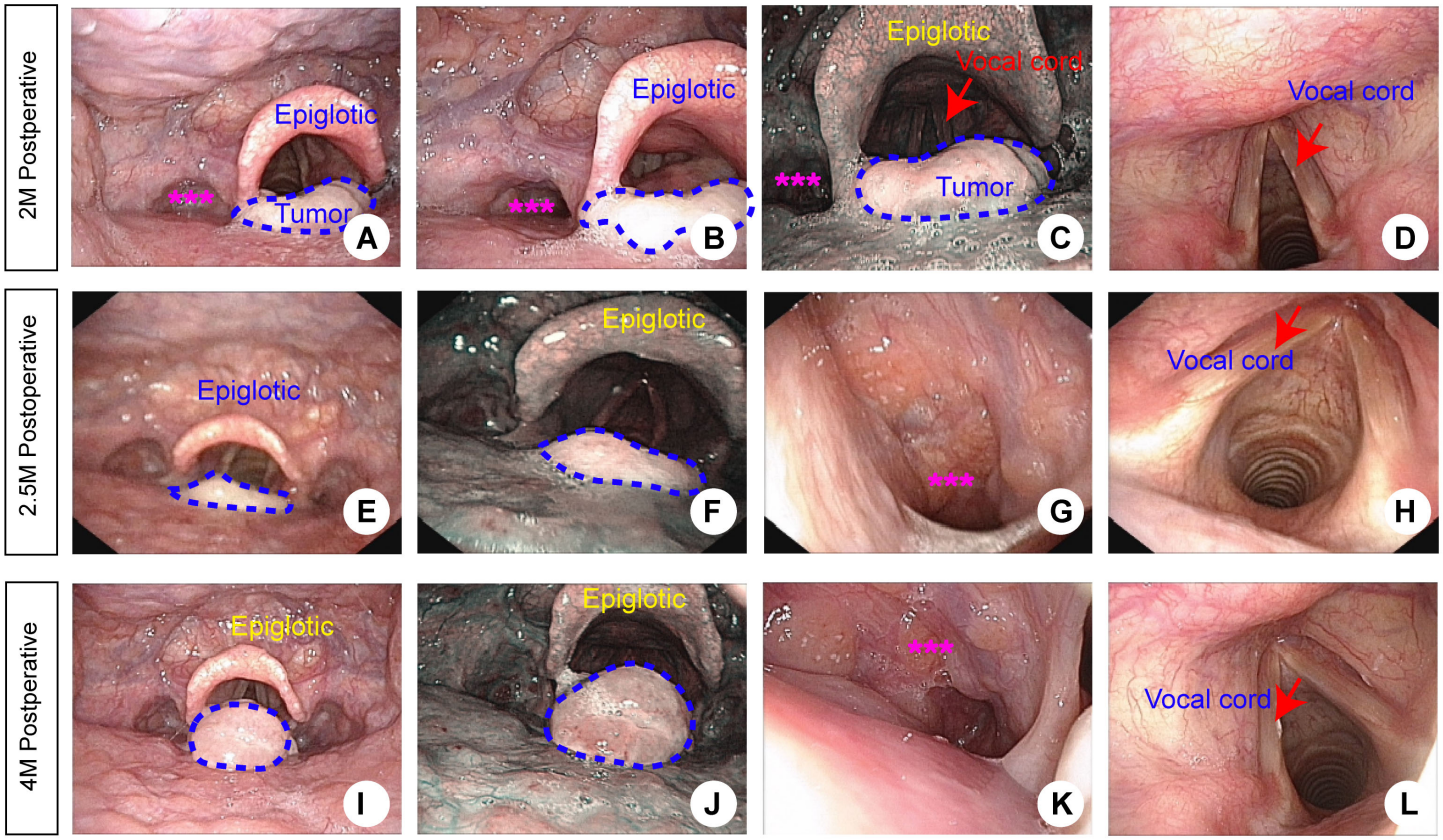
Serial postoperative laryngoscopic findings showing early local recurrence. At 2 months after surgery, white-light and narrow-band imaging views demonstrated a recurrent submucosal mass at the operative site in the left hypopharyngeal region, adjacent to the epiglottis, while vocal cord mobility was preserved **(A–D)**. At 2.5 months after surgery, repeat laryngoscopy showed persistence and enlargement of the lesion at the same site **(E–H)**. At 4 months after surgery, serial laryngoscopic examinations confirmed further local recurrence at the operative site, with the lesion clearly visible on both white-light and narrow-band imaging views **(I–L)**.

A contrast-enhanced MRI performed one month after surgery demonstrated complete removal of the lesion, with no evidence of residual disease (**Figures 2E–H**). Follow-up contrast-enhanced MRI at 4 months after surgery revealed a newly detected enhancing lesion at the operative site. Given the negative surgical margins and absence of definite residual disease on the one-month postoperative MRI, this finding was interpreted as early local recurrence. (**Figures 2I–L**). Flexible laryngoscopy at 2 months after surgery revealed a recurrent lesion at the operative site. Subsequent endoscopic examinations at 2.5 and 4 months confirmed persistent local recurrence (**Figure 4**).

Histopathological examination of the resected specimen confirmed recurrent well-differentiated liposarcoma. The patient recovered uneventfully after the second operation and remains under close endoscopic and radiological surveillance.

## Discussion

Well-differentiated liposarcoma (WDLPS), also termed atypical lipomatous tumor (ALT), is a low-grade adipocytic neoplasm characterized by slow growth, limited metastatic potential, and a marked tendency for local recurrence. Although liposarcoma is one of the most common soft tissue sarcomas in adults, involvement of the head and neck region is uncommon. Primary WDLPS arising in the hypopharynx is particularly rare, and its nonspecific clinical presentation may lead to initial misinterpretation as a benign submucosal lesion. (4, 8, 9).

The present case is noteworthy for several reasons. First, the patient was a young adult, whereas most previously reported hypopharyngeal or laryngopharyngeal ALT/WDLPS cases have occurred in middle-aged or elderly patients. Second, the lesion presented as a smooth, well-circumscribed submucosal mass without cervical lymphadenopathy, making it clinically difficult to distinguish from benign hypopharyngeal lesions. Third, despite apparently complete transoral excision and negative surgical margins, follow-up MRI at 2 months demonstrated a recurrent lesion in the hypopharyngeal region. The patient subsequently underwent repeat transoral excision at 5 months after the initial surgery, and histopathological examination confirmed recurrent WDLPS. This early recurrence highlights the need for close postoperative surveillance even when the initial excision appears complete.

## Comparison with previously reported cases

A review of PubMed-indexed reports shows that ALT/WDLPS involving the hypopharynx andlaryngopharyngeal region has been reported only rarely, mostly as isolated case reports or small case series (4, 5, 10–14). Previously reported lesions have most commonly involved the hypopharynx, particularly the pyriform sinus, as well as the supraglottic larynx (**Supplementary Table 1**). Clinically, these tumors often present as slowly enlarging submucosal or polypoid masses. Depending on tumor size and location, symptoms may include foreign body sensation, dysphagia, airway obstruction, voice change, or throat discomfort.

Compared with earlier reports, the most distinctive feature of the present case is the very short interval to recurrence. In many published cases, recurrence occurred months to years after the primary operation, reflecting the indolent but locally recurrent nature of WDLPS. In contrast, our patient developed radiologically detectable recurrence only 2 months after surgery, followed by pathological confirmation after re-excision at 5 months. This clinical course suggests that hypopharyngeal WDLPS may recur early even after macroscopically complete removal, possibly because of the narrow anatomical space, complex mucosal folds, and difficulty in achieving wide margins while preserving pharyngolaryngeal function.

## Early recurrence versus residual disease

The distinction between true recurrence and residual disease is important in the present case because the interval between initial surgery and postoperative detection of a lesion at the operative site was short. Several findings support early local recurrence in this patient. First, the primary tumor was removed en bloc, and no gross residual lesion was observed intraoperatively. Second, final histopathological examination showed negative surgical margins. Third, contrast-enhanced MRI performed one month after surgery demonstrated no definite residual disease. Finally, the lesion detected during follow-up was resected and pathologically confirmed as well-differentiated liposarcoma. Therefore, the postoperative lesion was interpreted as early local recurrence. However, because the hypopharynx is anatomically narrow and functionally important, wide margins are difficult to obtain, and the possibility of microscopic residual disease cannot be completely excluded. This consideration further supports the need for close postoperative endoscopic and radiological surveillance.

## Pathological and molecular characteristics

Histopathologically, WDLPS is characterized by mature adipocytes interspersed with atypical stromal cells and fibrous septa containing hyperchromatic nuclei, features that are considered diagnostic at the microscopic level (15) and further supported by classic descriptions of atypical lipomatous tumors/WDLPS morphology (16). The FISH analysis in this case confirmed MDM2 gene amplification, a defining molecular feature of WDLPS and the current gold standard for its molecular diagnosis, further supporting the diagnosis (17, 18). Taken together, these histomorphologic, immunohistochemical, and molecular findings are fully consistent with the diagnostic criteria recommended by the WHO 2020 classification of soft tissue and bone tumors.

## Treatment and recurrence

Complete surgical excision remains the mainstay of treatment for hypopharyngeal WDLPS/ALT. Because these tumors are rare and often arise in anatomically complex regions such as the hypopharynx, pyriform sinus, and supraglottic larynx, the surgical approach should be individualized according to tumor size, attachment, depth of extension, and relationship to surrounding structures. Previous clinicopathological series of laryngeal and hypopharyngeal liposarcoma have shown that surgical treatment is the principal therapeutic strategy, with local recurrence rather than distant metastasis representing the major clinical concern (2, 3).

For small and well-circumscribed lesions, transoral excision may provide adequate exposure while minimizing surgical morbidity. Recent reports of hypopharyngeal ALT/WDLPS also support the feasibility of transoral or endoscopic excision in selected patients, particularly when the lesion is pedunculated or well demarcated (19, 20). However, because the hypopharynx is anatomically narrow and functionally important, achieving wide margins can be difficult. This may partly explain the risk of local recurrence despite apparently complete excision.

In the present case, the tumor was removed transorally and the final pathology showed negative surgical margins. Nevertheless, recurrence was detected by MRI at 2 months and confirmed pathologically after repeat surgery at 5 months. This finding suggests that negative margins in hypopharyngeal WDLPS should be interpreted cautiously, especially when the lesion is close to complex mucosal folds or when the resection margin is limited by functional preservation. Early postoperative imaging and serial endoscopic examination are therefore important.

The role of adjuvant radiotherapy or chemotherapy remains uncertain for WDLPS. Because WDLPS has low metastatic potential, adjuvant treatment is generally not routinely required after complete excision. However, radiotherapy may be considered in selected cases with repeated recurrence, positive or close margins, unresectable disease, or dedifferentiated transformation. In this patient, the key management issue was early local recurrence rather than regional or distant spread, supporting repeat complete excision and close surveillance as the principal strategy.

## Clinical implications

This case provides several important clinical lessons. First, persistent foreign body sensation caused by a smooth hypopharyngeal submucosal mass should not be assumed to be benign, particularly when symptoms persist despite empirical treatment. Second, definitive diagnosis requires careful histopathological evaluation supported by MDM2/CDK4 immunohistochemistry and molecular confirmation of MDM2 amplification. Third, hypopharyngeal WDLPS can recur very early after surgery, even when the initial resection appears complete and margins are negative.

For follow-up, flexible laryngoscopy is useful for direct visualization of mucosal or submucosal recurrence, while MRI is valuable for assessing deeper extension and distinguishing postoperative changes from recurrent tumor. Based on the present case, close surveillance during the early postoperative period is particularly important. Long-term follow-up is also necessary because WDLPS is known for its potential for repeated local recurrence over extended periods.

This case has several limitations. First, the follow-up period remains relatively short, which precludes conclusions regarding long-term oncologic outcome. Given the known potential of WDLPS for repeated and late local recurrence, continued long-term endoscopic and radiological surveillance is required. Second, the foreign body sensation was not quantified using a validated symptom score. Third, although MRI was appropriate for soft-tissue assessment, additional imaging modalities were not performed because of the small and well-circumscribed nature of the lesion.

## Data Availability

The original contributions presented in the study are included in the article/**Supplementary Material**. Further inquiries can be directed to the corresponding author.

## Supplementary material

Supplementary Table 1 Reported cases of atypical lipomatous tumor/well-differentiated liposarcoma involving the hypopharynx/laryngopharyngeal region.
