# Supplementary material for: Early local recurrence of well-differentiated liposarcoma of the hypopharynx in a young adult: a case report and literature review
Source: Front Oncol. 2026 Jun 22;16:1877159. doi: 10.3389/fonc.2026.1877159 (PMC13333391; doi:10.3389/fonc.2026.1877159)
Supplement: Supplementary Table 1 — Reported cases of atypical lipomatous tumor/well-differentiated liposarcoma involving the hypopharynx/laryngopharyngeal region. [file Table1.docx]

## **Supplemental Table 1.** Previously reported well-differentiated liposarcoma/atypical lipomatous tumor involving the hypopharyngeal, pharyngeal, and laryngeal regions

| **First author, year** | **Study type** | **Site** | **Diagnosis / histology** | **Treatment** | **Follow-up / outcome** |
| --- | --- | --- | --- | --- | --- |
| Wening, 1990(Wenig et al., 1990) | Case series, 10 cases | Supraglottic larynx and hypopharynx, including pyriform sinus | Well-differentiated liposarcoma, grade I | Surgical treatment | No metastasis reported; multiple local recurrences were common |
| Wening,199(Wenig and Heffner, 1995) | Case series and literature review | Larynx and hypopharynx | Well-differentiated liposarcoma | Surgical treatment | Tendency for recurrence over extended periods |
| McQueen, 2010  (McQueen et al., 2010) | Case report | Hypopharynx | Giant atypical lipomatous tumor | Surgical excision | Follow-up not stated in PubMed abstract |
| Shi, 2010  (Shi et al., 2010) | Case series, 5 cases | Laryngopharynx; hypopharyngeal and laryngeal/epiglottic sites | Atypical lipomatous tumor | Surgical excision | Recurrence reported in 2 cases at 6 and 14 months |
| Nouri, 2011(Nouri et al., 2011) | Case report | Hypopharynx, right lateral wall | Well-differentiated liposarcoma | Surgical excision | Follow-up not stated in PubMed abstract |
| Zhu, 2017(Zhu et al., 2017) | Case series, 3 cases | Laryngeal / hypopharyngeal region | Well-differentiated liposarcoma with MDM2 amplification | Surgical excision | Local recurrence reported; no distant metastasis reported |
| Ioanidis, 2017 (Ioanidis et al., 2017) | Case report | Hypopharynx | Atypical lipomatous tumor / well-differentiated liposarcoma | Surgical excision | No recurrence at 22 months postoperatively |
| Fritchie, 2020 (Fritchie et al., 2020) | Case series, 8 cases | Upper aerodigestive tract: hypopharynx, larynx, and oral cavity | WDLPS / dedifferentiated liposarcoma | Surgical management | All cases with >1 year follow-up recurred at 45–118 months; one progressed to dedifferentiated liposarcoma |
| Al-Kadi, 2022 (Al-Kadi et al., 2022) | Case report | Hypopharynx / left pyriform sinus | Atypical lipomatous tumor with MDM2 amplification | Transoral endoscopic excision | No recurrence during nearly 2 years of follow-up; later died of unrelated cause |
| Dela Cruz, 2022 (Dela Cruz et al., 2022) | Case report | Oropharynx | Atypical lipomatous tumor / well-differentiated liposarcoma | Transoral excision followed by adjuvant radiotherapy | No evidence of disease at 6 months postoperatively |
| Murshed, 2022 (Murshed et al., 2022) | Case report | Posterior wall of hypopharynx | WDLPS with myxoid-like morphology; MDM2 and DDIT3 co-amplification | Surgical excision | Follow-up not clearly stated in accessible abstract |
| Kanaris, 2024 (Kanaris et al., 2024) | Case report | Hypopharynx | Well-differentiated liposarcoma confirmed by MDM2 amplification | Transoral blue laser excision | Follow-up not stated in PubMed abstract |
